# Supplementary material for: Clinical characteristics of sarcoma cases in which long-term disease control was achieved with trabectedin treatment: A retrospective study
Source: PLoS One. 2023 Mar 1;18(3):e0280508. doi: 10.1371/journal.pone.0280508 (PMC9977011; doi:10.1371/journal.pone.0280508)
Supplement: Attachment — Submitted filename: Response to Reviewers.docx [file pone.0280508.s002.docx]

**(Reviewer #1)**

**Comment 1**. This is a nicely written, albeit relatively small single-institution study evaluating the use of trabectedin in sarcoma. Overall, it provides useful, real-world information about the value of trabectedin long term in a subset of patients. This would be amplified if the authors pooled their data with one or more additional institutions. Since it was single-institution, there would theoretically be the opportunity to do additional tissue-based studies to look for some of the previously described tumor markers of interest. A few additional minor comments:

**Response**: We deeply appreciate the warm and suggestive comment.

**Comment 2**. Page 3, line 53- The authors mention that trabectedin is examined especially in translocation associated sarcoma, but do not explain the rationale. This should be clearly explained to the reader unfamiliar with the reasoning.

**Response**: Thank you for the comment. As suggested, we added one sentence explaining the rationale as follows:

(Line 53-55) This agent has been examined especially for translocation-related sarcoma because trabectedin mechanistically suppresses the transcription of the oncogenic fusion proteins such as FUS-DDIT.

**Comment 3**. Page 9- Since a main focus of this manuscript is the long-term patients, it would be helpful to include as much information as possible. Can the authors verify if patients had demonstrated progression prior to treatment? Amongst those with stable diseases, can a measurement of best tumor response/tumor shrinkage be included for these patients or a waterfall plot of all patients? As there are some patients with those histologies who can demonstrate an indolent course, in would be helpful to validate that there was benefit here.

**Response**: Thank you for the essential comment. At first, we verified that all patients experienced disease progression prior to trabectedin using CT. Secondly, we summarized the maximum tumor response/tumor shrinkage rate from the baseline in Table 2. Thirdly, subtypes in patients who achieved long-term disease control (PFS > 1-year) are shown in Table 2. Except for small round cell sarcomas such as Ewing sarcoma, tumor differentiation score 1-2 in FNCLCC grade might reflect an indolent clinical behavior. In the context, we consider that 4 patients (patient ID 1,3,4,5) with tumor differentiation score 2 are cases with histologies who can demonstrate an indolent course. In terms of actual tumor volume, patient ID-6 had a single lung metastasis with a diameter of about 9 mm, which indicates an indolent behavior.

(Line 160-161) All 6 patients had confirmed disease progression prior to trabectedin initiation using computed tomography (CT).

**Comment 4**. An additional reference that could be included was only available in abstract form but is relevant and could be added. Presented at CTOS: Davis EJ, et al. Abstract 277. Presented at: Connective Tissue Oncology Society Annual Meeting; Nov. 8-11, 2017; Maui.. Also presented at ESMO: Annals of Oncology (2017) 28 (suppl_5): v521-v538. 10.1093/annonc/mdx387

**Response**: Thank you for the informative comment. We cited the reference at the part of discussion as follows:

(Line 217-224) Davis et al. retrospectively compared clinical outcomes between 268 patients with sarcoma who received trabectedin for 6-12 months and 133 patients who received the treatment over 12 months. Reported ORR and median OS in the both treatment groups were 8% and 7%, and 18.1 months and 47.0 months, respectively. Two patients (1 with synovial sarcoma and 1 with uterine leiomyosarcoma) underwent the treatment over 50 months. As with our data, patients who required cycle delay and dose reduction accounted for 62% and 78% of > 12 months group.

**Comment 5**. The discussion section is very nicely written in pointing out the limitations of this analysis as well as the value of trabectedin in a subset. Congratulations to the authors.

**Response**: Thank you so much for the kind comment.

**Comment 6**. Table 2, typo in legend- overall survival

**Response**: Thank you for the comment. We corrected the word in legend.

**Comment 7**. Figure 1 is slightly blurry in reproduction.

**Response**: Thank you for the comment. We corrected Figure 1.

**(Reviewer #2)**

**Comment 1.** Based on the Results section and Table 1, common histologies (myxoid liposarcoma, synovial sarcoma, leiomyosarcoma, and dedifferentiated liposarcoma) included 34 (67%) of the 51 patients. Would it be possible to report the rest as “other” or provide the other sarcomas as a foot note for Table 1?

**Response**: Thank you for the comment. As suggested, we added chondrosarcoma (n=4) and undifferentiated pleomorphic sarcoma (n=3) to the list in Table 1, and described the detailed histology information in a foot note as follows:

*Other histology: alveolar soft part sarcoma (n=2), endometrial stromal sarcoma (n=2), rhabdomyosarcoma (n=1), Ewing sarcoma (n=1), clear cell sarcoma (n=1), spindle cell sarcoma (n=1), undifferentiated endometrial sarcoma (n=1), undifferentiated sarcoma (n=1)

**Comment 2.** If 51 patients were treated, why do we only have FNCLCC grades for 37 of 51 patients listed in the first paragraph of the Results section and on Table 1?

**Response**: Thank you for the great comment. FNCLCC grades in our study were based on the pathology report in respective patient. Unfortunately, they were unavailable in 14 patients. Regarding to 3 out of the 14 patients, Japanese grade composed of tumor differentiation, tumor necrosis and Ki-67 index (not mitotic count) was alternatively obtained (Hum Pathol 2002; 33: 111-5). Two patients had grade 3 and 1 had grade 2. We clarified this point in the limitation, and gave an additional explanation in a foot note.

(Line 259-261) Furthermore, FNCLCC grades in our study were based on the pathology report in respective patient, and they were unavailable in 14 patients (27%).

**Japanese grade composed of tumor differentiation, tumor necrosis and MIB-1 index was available in other 3 patients. Grade 2 (n=1), grade 3 (n=2)

**Comment 3.** If 51 patients were treated and 26 had fusions, shouldn’t the percentage be 51% and not 53%? Is the 53% due to the number of evaluable patients (n=49), as stated in the Clinical outcomes section? It may be useful to clarify this point.

**Response**: Thank you for the comment. We corrected the percentage to 51% (26/51) in the main text and Table 1.

**Comment 4.** I assume that GEISTRA score was only assessed for 80% of patients based on the results section and Table 1 (Score 0 n=18, Score 1 n=23). Is this correct? Was there missing information for the remaining 20%?. It’s okay if some did not have sufficient information, but it would be good for this information to be clearly stated in the results section.

**Response**: Thank you for the comment. I am sorry for the confusion. We checked the original data again. As written in line 111-112, GEISTRA scores were 0 (n=18), 1 (n=23), and 2 (n=10). There were no cases with GEISTRA score 3. We added the missing information in Table 1.

**Comment 5.** In the Clinical outcomes section, line 142 states “…among cases with GEISTRA scores 1, 2, and 3…” The results section/table 1 does not include any numbers for GEISTRA score 3.

**Response**: Thank you for the comment. As we responded to comment 4, there were no cases with GEISTRA score 3. Therefore, we corrected the expression in line 142 as follows:

“among cases with GEISTRA scores 0, 1, and 2 (*P*= 0.61)”

**(Journal Requirements)**

1. Please ensure that your manuscript meets PLOS ONE's style requirements, including those for file naming.

**Response**: We modified our manuscript to PLOS ONE's style.

2. Please provide additional details regarding participant consent. In the Methods section, please ensure that you have specified (1) whether consent was informed and (2) what type you obtained (for instance, written or verbal). If your study included minors, state whether you obtained consent from parents or guardians. If the need for consent was waived by the ethics committee, please include this information

**Response**: We described additional details regarding participant consent.

(Line 75-76)

Informed consent of each patient was waived under the approval of the institutional review board.

3. In your Data Availability statement, you have not specified where the minimal data set underlying the results described in your manuscript can be found. PLOS defines a study's minimal data set as the underlying data used to reach the conclusions drawn in the manuscript and any additional data required to replicate the reported study findings in their entirety.

**Response**: We added my data availability statement in line 278-279.
