# Supplementary material for: Clinical characteristics of sarcoma cases in which long-term disease control was achieved with trabectedin treatment: A retrospective study
Source: PLoS One. 2023 Mar 1;18(3):e0280508. doi: 10.1371/journal.pone.0280508 (PMC9977011; doi:10.1371/journal.pone.0280508)
Supplement: Attachment — Submitted filename: Response to Reviewers.docx [file pone.0280508.s003.docx]

**(Reviewer #1)**

**Comment 1**. The authors addressed comments and once again put forward an informative manuscript. My only remaining comment is more general. While the article relays good information, certain stylistic concerns remain in a scientific paper. Please consider having article briefly edited for scientific English language to improve on conciseness to better communicate the authors' points. It is noted that a program was used for this purpose, but a native speaker may provide more assistance.

**Response**: Thank you for the informative comment. We consulted with the professional English proofreading company again, and a native English editor modified throughout my original manuscript.

**Comment 2**. Page 3, line 66; "It is very important to predict who can continue treatment sufficiently before trabectedin is administered; yet, there is insufficient clinical information regarding this point." could easily be adjusted to "Despite the importance, there are few known clinical predictive factors of benefit to trabectedin" or something like that.

**Response**: Thank you for the comment. As suggested, we corrected the original sentence (Page 3, line 64).

Despite its importance, few clinical predictive factors of trabectedin’s benefits are known.

**Comment 3**. Page 13, line 247, "Originally, a specific translocation (EWSR1-FLI1) could be the target of trabectedin, and further investigations are awaited [24]". This line could be adjusted minimally to enhance understanding to "While EWSR1-FLI1 was originally hypothesized to be a potential target for trabectedin, this has not been seen clinically and more studies are needed" or something similar.

**Response**: Thank you for the comment. As suggested, we corrected the original sentence (Page 13, line 240-241).

Although EWSR1-FLI1 was originally considered a potential target for trabectedin, this has not been verified clinically, and further research is required.

**Comment 4**. Finally, would suggest minor adjustments to Conclusions. Currently reads as " This study suggests that trabectedin continuation at a sustainable dose and interval might be clinically beneficial in some patients with a focus on L-sarcomas. Specifically, a longer duration is expected for myxoid liposarcoma since the tumor shrinkage effect is generally slow." This may better relay the author's main points as "The study suggests that trabectedin continuation at a sustainable dose and interval is clinically beneficial in a subset of sarcoma patients, even if substantially lower dosing and longer dosing intervals than current standards. The time to tumor shrinkage is relatively long, particularly in patients with myxoid liposarcoma."

**Response**: Thank you for the great comment. As suggested, we corrected the original sentence (Page 14, line 258-261).

Trabectedin continuation at a sustainable dose and interval is clinically beneficial in a subset of sarcoma patients, even at substantially lower doses and longer dosing intervals than the current standards. The time to tumor shrinkage is relatively long, particularly in myxoid liposarcoma patients.

**Comment 5**. There are several other instances where a quick read through to adjust language can help with readability. Beyond that, the authors have done an excellent job trying to relay their work..

Thank you for the comment. A native English editor modified throughout my original manuscript. These points are highlighted in yellow. We attach Certificate of Proofreading as a supplementary document.
